# Headland on the Action of Medicines in the System

**Published:** 1853-07

**Authors:** 


*Headland on the Action of Medicines in the System.* Philadelphia. Lindsay & Blakiston, 1853.

This treatise is the one to which the Fothergillian gold medal for 1852 was awarded. It is conceived in an admirable spirit of candor, and is written in a severely logical style. The author lays down certain propositions which he discusses in regular order, after having first taken a general survey of the subject and given a brief synopsis of the views entertained by other authors respecting the action of medicines and the proper method of classifying them.

The author maintains the opinion that the great majority of medicines must obtain admission into the blood before their specific influence can be exhibited. He establishes this by numerous experiments of different observers, and so fortifies his position as to leave little chance for a reply.

He then proceeds to show how they get admission into the blood, and concludes that those which are not dissolved by the liquids of the stomach, such as the resinous substances, undergo a digestion in the intestinal canal and are absorbed by the veins coming off from that mucous expansion. Under this head, he considers the recently broached *endosmotic theory of saline purgatives,* and shows its fallacy by experiments on living animals as well as on dead animal membrane. While considering the digestibility of medicines, he makes use of Bernard's theory of the action of the pancreatic juice to account for the digestion of the oily medicines. Now, independently of the doubt which has been cast both by Frerichs and Schumann upon this theory, it is impossible to believe that the oily purgatives, for example, must be digested before they act upon the bowels. Independently of the difficulty attendant upon the digestion and absorption of such quantities of fat, we know that some of these agents have been detected little changed, either in quality or quantity, in the dejections which they produce.

As a corollary to the last proposition, it follows that those medicines which are insoluble in water, and in gastric and intestinal juice, cannot get into the circulation and must therefore act locally. Under this head, he is compelled to notice the experiments of Œsterlen of Dorpat, who asserts that he has seen solid particles of charcoal, varying from 1-6000 to 1-3000 of an inch in diameter taken up by the portal vein, and that he has found minute globules of mercury under the skin, after rubbing with mercurial ointment. Dr. Headland repeated these experiments with different substances, but never could detect any traces of such absorption.

This leads to the consideration of the remedial agents which act without being absorbed. These the author considers to be *irritant emetics,* such as sulphate of zinc, mustard, &c., *stomach anæsthetics,* as nitrate of bismuth, and *irritant cathartics,* as scammony, gamboge, &c., as well as the mechanical anthelmintics. He shows very clearly the difference between an irritant and a specific emetic or cathartic.

A necessary consequence of the propositions already laid down is, that the medicines, when in the blood, must permeate the mass of the circulation, so far as may be required to reach the parts on which they tend to act, the only exception to this rule being those cases in which either sensation, pain or muscular contraction are produced as a distinct point.

Having thus established the passage into the circulatory fluid of most of the medicines we introduce into the stomach, the author proceeds to examine the method in which they act upon the system through the medium of the blood. The changes they produce in this fluid he classifies under three heads: *first,* changes in combination, as when an alkali which will neutralize an excess of acid in the blood, will diminish the elimination of that acid in the urine; *secondly,* changes of reconstruction, as when benzoic and cinnamic acids are changed into hippuric acid; and *thirdly,* changes of decomposition, as when lactate of soda is oxydized to carbonate of soda.

Medicines acting upon the blood itself, he calls *hæmatics,* and supposes that they produce permanent changes in that fluid. These may be either *restorative,* supplying or causing to be supplied a deficient material, as when iron or dried bullock's blood is administered in anæmia, or *catalytic,* counteracting a morbid process and passing out of the body with the results of their action, as when mercury destroys the poison of syphilis, if, indeed, this action ever takes place.

Medicines which pass out of the blood to the nerve-centres, he calls *neurotics,* and classifies under this head, *stimulants, sedatives* and *narcotics. Astringents* he supposes to pass from the blood to the muscular fibre and *eliminatives* to have a special tendency towards the glands, exciting them to action, as they pass through them.

These different propositions are argued out with great skill and uncommon fairness. We must, however, take exceptions to one or two of our author's positions, while, at the same time, we fully acknowledge the benefit which this little treatise must confer upon scientific therapeutics.

He supposes, (to illustrate our objections, for we do not mean to go into detail,) that the oxydation of the body is increased during febrile diseases, a doctrine borrowed from Liebig. A deficiency of lactic acid is the consequence, because the decomposition of the tissues tends rather to the production of the highly nitrogenous acids. If then the vegetable acids are introduced, substances containing more oxygen get into the circulation, so that less is required for their metamorphosis, and consequently less heat is generated. Lehmann, however, has made sad havoc with this theory by shaking the substratum on which it rested. He has shown that there is actually less oxydation during febrile diseases, than at other times, and that the true account of those supposed signs of oxydation, is to say that the balance between circulation and respiration is lost.

So, too, the interpretation of the benefit of cod liver oil to phthisical patients, based upon Liebig's oxydation theory, must be abandoned when we learn that in phthisis, oxydation is also retarded. Lehmann's idea that the fat of the oil serves as a sort of nucleus round which nutrition can begin, is much more reasonable.

Setting aside these, however, which are merely minor defects, and do not affect the real merits of the work, we heartily commend it as a philosophical treatise and an effort in the right direction, which we shall be glad to see followed up by others.

